# Congenital bronchogenic cyst: A case study on early detection and surgical intervention

**DOI:** 10.1016/j.ijscr.2025.111120

**Published:** 2025-03-04

**Authors:** Janaa Manasrah, Ahmad Fasfoos, Maaweya Jabareen, Wasef Alhroub, Yousef Abu Asbeh

**Affiliations:** a Faculty of Medicine, Hebron University, Hebron, Palestine; b Al-Ahli Hospital, Hebron, Palestine

**Keywords:** Bronchogenic cyst, Thoracic surgery, Video-assisted thoracoscopic surgery (VATS), Pediatric cystic lung lesions

## Abstract

**Introduction:**

The patient showed an uneventful recovery post-surgery, underscoring the significance of early intervention in managing rare congenital anomalies. Bronchogenic cysts constitute 13–15 % of congenital cystic lung diseases and 6 % of childhood mediastinal masses. Arising from abnormal foregut development during embryogenesis, these cysts can be asymptomatic or cause serious complications, such as airway or vascular compression.

**Case presentation:**

A 10-day-old female newborn presented with a pulmonary cystic lesion in the left upper lung lobe, identified via routine antenatal ultrasound. She was asymptomatic at birth, with no respiratory distress or congenital anomalies. Chest CT revealed a large bilocular cyst causing lung compression and mediastinal shift. Initial diagnosis suggested Congenital Pulmonary Airway Malformation (CPAM) Type 1. Video-assisted thoracoscopic surgery (VATS) successfully removed the lesion, with histopathology confirming a bronchogenic cyst.

**Discussion:**

Bronchogenic cysts result from abnormal budding of the foregut during embryogenesis and typically present as unilocular cysts without communication with the bronchial tree. While often asymptomatic, these cysts can cause life-threatening complications due to compression effects. Imaging modalities, including prenatal ultrasonography, fetal MRI, and postnatal CT scans, are crucial for diagnosis. Histopathology provides confirmation by identifying the characteristic ciliated pseudostratified columnar epithelium. Differential diagnoses, such as CPAM or lung sequestration, must be ruled out. Surgical excision is the gold standard treatment to prevent complications like infection, hemorrhage, or malignant transformation.

**Conclusion:**

This case underscores the importance of early diagnosis and prompt surgical management of bronchogenic cysts. Timely intervention ensures a successful recovery and prevents life-threatening complications, even in asymptomatic infants.

## Highlights

• Bronchogenic cysts are rare congenital anomalies resulting from abnormal bronchial budding.
• The bronchogenic cysts typically are asymptomatic but may become symptomatic due to the mass effect.
• Definitive diagnosis of bronchogenic is obtained by histopathological examination after surgical excision.
• Surgical resection is the gold standard treatment for bronchogenic cysts

## 1 Introduction

This case report has been prepared in compliance with the SCARE 2023 guidelines for surgical case reporting [1].

Bronchogenic cysts are rare congenital anomalies resulting from abnormal bronchial budding during early embryogenesis [2,3]. These cysts account for approximately 13 % to 15 % of congenital cystic lung diseases and 6 % of mediastinal masses in children [4]. Most bronchogenic cysts are found in the middle mediastinum, although they can also occur in unusual locations such as the lung parenchyma, head, neck, or abdomen [5].

Symptoms usually arise from compression effects or infection. While infants and children patients often present with compressive symptoms, infections are more common in adults [6,7]. In some cases, these cysts may remain asymptomatic [7]. The standard treatment for BCs is complete surgical excision, with histopathological examination being the primary method for establishing a definitive diagnosis [8].

This case present case of a 10-day-old female newborn, identified antenatally with a pulmonary cystic lesion in the left upper lung lobe, the newborn was asymptomatic at birth, with no respiratory distress or other congenital defects.

## 2 Case presentation

A 40-year-old female, gravida 5, para 4, delivered a 2900 g female child via cesarean section at 35 + 6 weeks of gestation. The delivery was uncomplicated until a large pulmonary cystic lesion in the left upper lung lobe was noted, previously identified through antenatal ultrasonography. At birth, the child exhibited stable vitals with a respiratory rate of 42, a pulse rate of 130, an oxygen saturation of 94 %, and an initial APGAR score of 8. However, within minutes postdelivery, mild respiratory distress was noted, which resolved spontaneously without intervention. Antenatal ultrasounds conducted during the sixth and ninth months of pregnancy reported normal fetal organ development except for the bronchial congenital defect identified shortly before delivery. The amniotic fluid index remained within normal limits throughout the pregnancy. The mother's pregnancy was largely uneventful, aside from a brief episode of pneumonia in the seventh month, treated effectively with antibiotics. Her medical history included polycystic ovary syndrome and a history of fertility treatments.

**Fig. 1:**
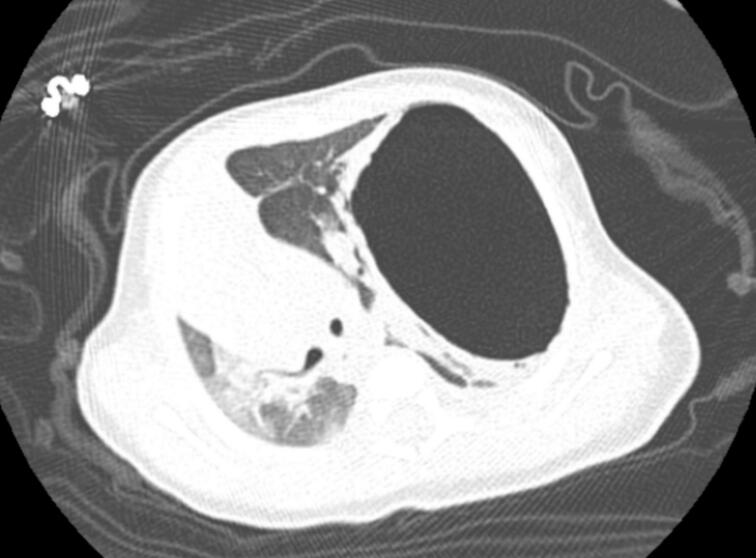
CT scan show there is a large bilocular cystic lesion measuring about 3.5 ∗ 5.5 ∗ (TS ∗ AP), occupying left upper lung lobe associated with surrounding consolidative and ground glass opacities causing a contralateral mediastinal and tracheal shift associated with diffuse bilateral mosaic attenuation along with another small cavitary cystic lesion measuring about 2 mm in diameter. No visible communication with the bronchial system.

On clinical examination, air entry was diminished in the left lung, and cardiovascular examination showed tachycardia with a regular rate and rhythm. A chest CT scan with IV contrast on the third day of life showed a large, bilocular cystic lesion in the left upper-mid lung lobe measuring 3.5 × 5.5 cm, with cavitation, fluid-air levels, and thick enhancing walls (Fig. 1), The CT scan we performed with sedation with midazolam per the hospital protocol for this age group.

The lesion extended into the posterior mediastinum, causing significant compression of the underlying lung parenchyma, compressive atelectasis, and a contralateral mediastinal and tracheal shift, along with smaller cavitary cystic lesions and diffuse bilateral mosaic attenuation. Based on the imaging and clinical status, a decision was made to insert a left pigtail catheter and consider video-assisted thoracoscopic surgery (VATS) if necessary.

**Fig. 2:**
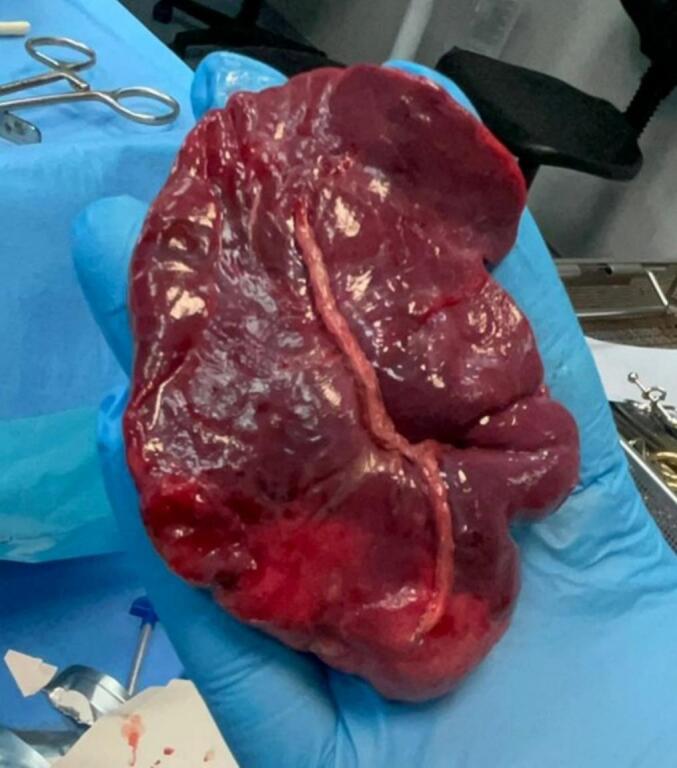
Photograph of the gross bronchogenic cyst specimen.

**Fig. 3:**
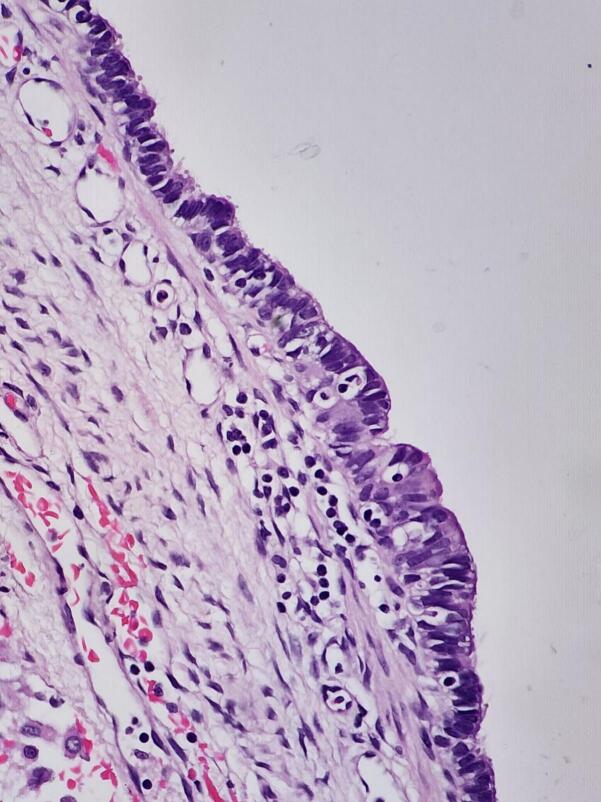
Bronchogenic cyst; sections revealed unilocular cyst lined by bland respiratory type epithelium. The underlying cyst lung parenchyma is congested.

The patient due to the huge size of the cyst underwent a left thoracotomy and left upper lobectomy. A large cystic lesion intraoperatively was seen compressing the left lower lobe. Wedge resection was done to get a good view of it followed by dissection and double ligation of left upper lobe vein and pulmonary artery branches. Excision of the cyst was done (Fig. 2) and sent for histopathology which proved to be a bronchogenic cyst (Fig. 3).

The patient was managed postoperatively in the PICU for one day and then transferred to the ward. The chest tube was removed on day five, and follow-up chest X-rays showed satisfactory lung expansion. The infant was discharged one week after surgery in stable condition with no respiratory complications, feeding well, and showing an uneventful recovery.

## 3 Discussion

Bronchogenic cysts are congenital respiratory tract cystic malformations caused by aberrant bronchial tree budding of the primitive foregut during embryogenesis [3,4]. The cysts consist of a thin, fibrous capsule lined with the secretory respiratory epithelium (columnar or cuboidal ciliated epithelium), which may also contain cartilage, smooth muscle, elastic tissue, and glandular tissue, which is similar to normal bronchial tissue. Most of the cysts are found along the tracheobronchial tree in the mediastinum, but they can also be found in the lung parenchyma or, in the case of dumbbell cysts, extend to or below the diaphragm [4,5,9].

The bronchogenic cysts typically are asymptomatic but may become symptomatic due to the mass effect [10]. Symptoms can appear in any age group due to compression of mediastinal airways, vascular structures, or esophagus. Usual symptoms include respiratory distress, cough with or without wheezing, repeated infections, dyspnea, dysphagia, arrhythmias, compression of vena cava, hemoptysis, hemothorax, and pneumothorax [11]. In our case, even though the infant was asymptomatic at birth, the possible life-threatening complications as Pneumothorax, Superior vena cava syndrome, Stenosis of the pulmonary artery, and Air embolism warranted early surgical intervention.

The diagnosis and histopathology of congenital bronchogenic cysts are somewhat different in the prenatal versus the postnatal period. The usual prenatal diagnosis is by detailed ultrasonography, and sometimes fetal MRI can reveal cystic lesions in the thorax in the second trimester onwards [12]. Most of the cysts are confirmed postnatally by imaging studies such as CT or MRI [13]. CT scans are essential for differentiating bronchogenic cysts from other congenital anomalies like CPAM and lung sequestration [14]. This was further supported by the fact that there was a cystic lesion with enhancing walls and no communication with the bronchial tree.

Definitive diagnosis of bronchoginic is obtained by histopathological examination after surgical excision [13]. Histologically, bronchogenic cysts are lined with ciliated pseudostratified columnar epithelium and may contain cartilage, smooth muscle, and glands, compatible with their origin from foregut anomalies. These findings confirm their congenital nature and exclude other cystic lung lesions [4].

Differential diagnoses for a congenital bronchogenic cyst include Congenital Pulmonary Airway Malformation (CPAM) with cystic or solid lesions and Lung Sequestration, a non-functioning lung segment with its own blood supply [15,16]. It is important for them to be accurately diagnosed and treated with accurate imaging and assessment.

Our review identified few documented cases of fetus with Prenatal Detection and Postnatal Evaluation of Bronchogenic Cysts. One such case, reported in 2013, was a prenatal diagnosis of asymptomatic bronchogenic cyst confirmed in the postnatal period by CT [17]. Another case, reported in 2024, was a fetus prenatal diagnosis of bronchogenic cyst experiencing compression symptoms and confirmed diagnosis by histopathological examination after successful excision of the cyst [13]. Another case, reported in 2000, a fetus was prenatally diagnosed with an asymptomatic bronchogenic cyst, with the diagnosis confirmed through histopathological examination after successful cyst excision [18]. Another case, reported in 2021, was a prenatal diagnosis of asymptomatic bronchogenic cyst During the COVID-19 pandemic confirmed in the postnatal period by pathological findings [12]. And finally, a case reported in 2021 of a prenatal diagnosis of enlarged bronchogenic cyst causing hydrops fetalis was confirmed with fetal MRI [19].

In our case, a 10-day-old girl was diagnosed with a cystic lesion in the left upper lobe, first detected during routine antenatal ultrasonography. The remainder of the congenital anomaly scan was unremarkable, and the baby delivered without any respiratory distress. In the absence of symptoms, a chest CT was undertaken postnatally, which showed a large, bilocular cystic lesion of the left upper and midlung lobe measuring approximately 3.5 × 5.5 × 5.5 cm. The lesion had thickened, enhancing walls with a fluid-air level, significant compression of the underlying lung parenchyma with associated atelectasis, and contralateral mediastinal and tracheal shift. A complicated cyst was suggested.

Given the high degree of compression and the complications that may arise, a left thoracotomy and upper lobectomy were performed 10 days later. A large, overinflated cystic lesion causing significant compression of the left lower lobe was intraoperatively identified. After meticulous dissection and division of vascular structures to the left upper lobe, the cystic lesion was excised and sent for histopathological evaluation. The results confirmed the diagnosis of a bronchogenic cyst.

Surgical resection is the gold standard treatment for bronchogenic cysts since it eliminates the risk of infection, hemorrhage, and malignant transformation [[20], [21], [22]]. In asymptomatic cases, elective resection may be considered, whereas symptomatic or complicated cysts require prompt intervention [22]. Studies have demonstrated the effectiveness of both VATS and open surgery in treating mediastinal or pulmonary Bronchogenic cyst. However, open surgery remains more use in cases involving large lesions [23]. Here, the patient had an uneventful surgical resection, and his postoperative course was unremarkable. Follow-up chest X-rays performed daily showed good lung re-expansion, and the chest tube was removed five days following surgery. The infant was stable, tolerated oral feeds well, and was discharged in good condition one week after surgery.

## 4 Conclusion

This case thus underlines the importance of early diagnosis by detailed ultrasonography at prenatal period or postnatally by imaging studies such as CT or MRI and timely intervention in congenital pulmonary malformations to prevent complications such as infection, hemorrhage, and malignant transformation. Though most bronchogenic cysts are asymptomatic, close follow-up and timely surgical intervention are necessary to avoid complications and achieve the best results. The postoperative course in our patient has been smooth, with the infant being asymptomatic, active, and accepting feeds well, which speaks to the success of early surgical intervention.

## Author contribution

J.M. handled conceptualization, data curation, and the writing of the original draft. A.F. writing: review, editing, and software. M.J. contributed to the investigation and visualization. W.A. managed resources and validation. Y.A. provided supervision.

## Informed consent

Written informed consent was obtained from the patient for publication of this case report and accompanying images. A copy of the written consent is available for review by the Editor-in-Chief of this journal on request.

## Ethical approval

Ethical approval was not applicable for this study, as our institution's IRB committee at Hebron University does not mandate approval for reporting individual cases or case series.

## Guarantor

Ahmad Fasfoos.

## Research registration number

Not applicable.

## Funding

This research did not receive any specific grants from funding agencies in the public, commercial, or not-for-profit sectors.

## Conflict of interest statement

The authors assert that no conflicts of interest exist in relation to this work.

## Data Availability

All data supporting the study's findings are included in the article and are readily accessible.

## References

[1] Sohrabi C., Mathew G., Maria N., Kerwan A., Franchi T., Agha R.A. (May 1 2023). The SCARE 2023 guideline: updating consensus Surgical CAse REport (SCARE) guidelines. Int. J. Surg. [Internet].

[2] Mampilly T., Kurian R., Shenai A. (2005). Bronchogenic cyst - cause of refractory wheezing in infancy. Indian J. Pediatr. [Internet].

[3] Whooley J., White A., Soo A. (Apr 4 2022). Bronchogenic cyst: a rare case of malignant transformation. BMJ Case Rep. [Internet].

[4] Sharma S., Limaiem F., Collier S.A., Mlika M. (Nov 9 2024). Bronchogenic cyst. StatPearls [Internet].

[5] Jiang J.H., Yen S.L., Lee S.Y., Chuang J.H. (2015 Mar 1). Differences in the distribution and presentation of bronchogenic cysts between adults and children. J. Pediatr. Surg. [Internet].

[6] Ribet M.E., Copin M.C., Gosselin B. (May 1 1995). Bronchogenic cysts of the mediastinum. J. Thorac. Cardiovasc. Surg..

[7] Davenport M., Warne S.A., Cacciaguerra S., Patel S., Greenough A., Nicolaides K. (2004). Current outcome of antenally diagnosed cystic lung disease. J. Pediatr. Surg. [Internet].

[8] Gross D.J., Briski L.M., Wherley E.M., Nguyen D.M. (Sep 25 2023). Bronchogenic cysts: a narrative review. Mediastinum [Internet].

[9] Singh A., Mandelia A., Kawdiya A., Naranje K., Nigam N. (Jan 1 2023). Evolution of antenatally diagnosed bronchogenic cyst in an infant. J. Indian Assoc. Pediatr. Surg. [Internet].

[10] Moremi M.D., Motene A.L., Maligavhada N.J., Tiva N.G., Mamogale R.T., Risenga S.M. (2018). A bronchogenic cyst masquerading as asthma: a case report. Afr. J. Thorac. Crit. Care Med. [Internet].

[11] Rahman S.M.T., Islam M.M., Akhter K.M., Islam M.Z., Hossain M. (Jan 1 2023). Bronchogenic cyst at unusual location. Respir. Med. Case Rep. [Internet].

[12] Cheng L., Duan J., Wang M., Lu D., Li H., Ma J. (Jul 7 2021). Case report: prenatal and postnatal management for fetal Bronchogenic cysts during the COVID-19 pandemic. Front. Pediatr. [Internet].

[13] Hsieh C.J., Huang S.Y., Chou C.M., Tseng J.J. (Mar 1 2024). Congenital bronchogenic cyst: from prenatal diagnosis to postnatal management. Taiwan. J. Obstet. Gynecol..

[14] Cancemi G., Distefano G., Vitaliti G., Milazzo D., Terzo G., Belfiore G. (Jun 1 2024). Congenital lung malformations: a pictorial review of imaging findings and a practical guide for diagnosis. Children [Internet].

[15] David M., Lamas-Pinheiro R., Henriques-Coelho T. (Aug 5 2016). Prenatal and postnatal management of congenital pulmonary airway malformation. Neonatology [Internet].

[16] Takei K., Takanashi Y., Shibata M., Sekihara K., Funai K., Takei K. (Nov 4 2024). Mediastinal extralobar pulmonary sequestration concurrent with spontaneous pneumothorax: a case report of difficult preoperative diagnosis. Cureus [Internet].

[17] Rios L.T.M., Júnior E.A., Nardozza L.M.M., Moron A.F., Martins M. de G. (Jan 1 2013). Prenatal diagnosis and postnatal findings of bronchogenic cyst. Case Rep. Pulmonol. [Internet].

[18] Bagolan P., Bilancioni E., Nahom A., Trucchi A., Inserra A., Neri M. (2000). Prenatal diagnosis of a bronchogenic cyst in an unusual site. Ultrasound Obstet. Gynecol. [Internet].

[19] Arnaoutoglou C., Spyrakos S., Kapetanaki A., Keivanidou A., Machairiotis N., Zarogoulidis P. (Jan 1 2021). Perinatal management of enlarged bronchogenic cyst causing hydrops fetalis. Respir Med Case Reports..

[20] Kirmani B., Kirmani B., Sogliani F. (Nov 1 2010). Should asymptomatic bronchogenic cysts in adults be treated conservatively or with surgery?. Interact. Cardiovasc. Thorac. Surg. [Internet].

[21] Aktoǧu S., Yuncu G., Halilçolar H., Ermete S., Buduneli T. (Oct 1 1996). Bronchogenic cysts: clinicopathological presentation and treatment. Eur. Respir. J. [Internet].

[22] Fievet L., Gossot D., de Lesquen H., Calabre C., Merrot T., Thomas P. (Nov 1 2021). Resection of bronchogenic cysts in symptomatic versus asymptomatic patients: an outcome analysis. Ann. Thorac. Surg. [Internet].

[23] Wang G., Ji X., Yao C., Jiang Y., Wang L., Li C. (May 31 2024). Comparative outcomes of video-assisted thoracoscopic surgery versus open surgery for bronchogenic cysts in adults: a retrospective cohort study. J. Thorac. Dis. [Internet].

